# Supplementary material for: Structural insights into cGAMP degradation by Ecto-nucleotide pyrophosphatase phosphodiesterase 1
Source: Nat Commun. 2018 Oct 24;9:4424. doi: 10.1038/s41467-018-06922-7 (PMC6200793; doi:10.1038/s41467-018-06922-7)
Supplement: Supplementary file 1 — Supplementary Information [file 41467_2018_6922_MOESM1_ESM.pdf]

Supplementary Information

Kato et al.

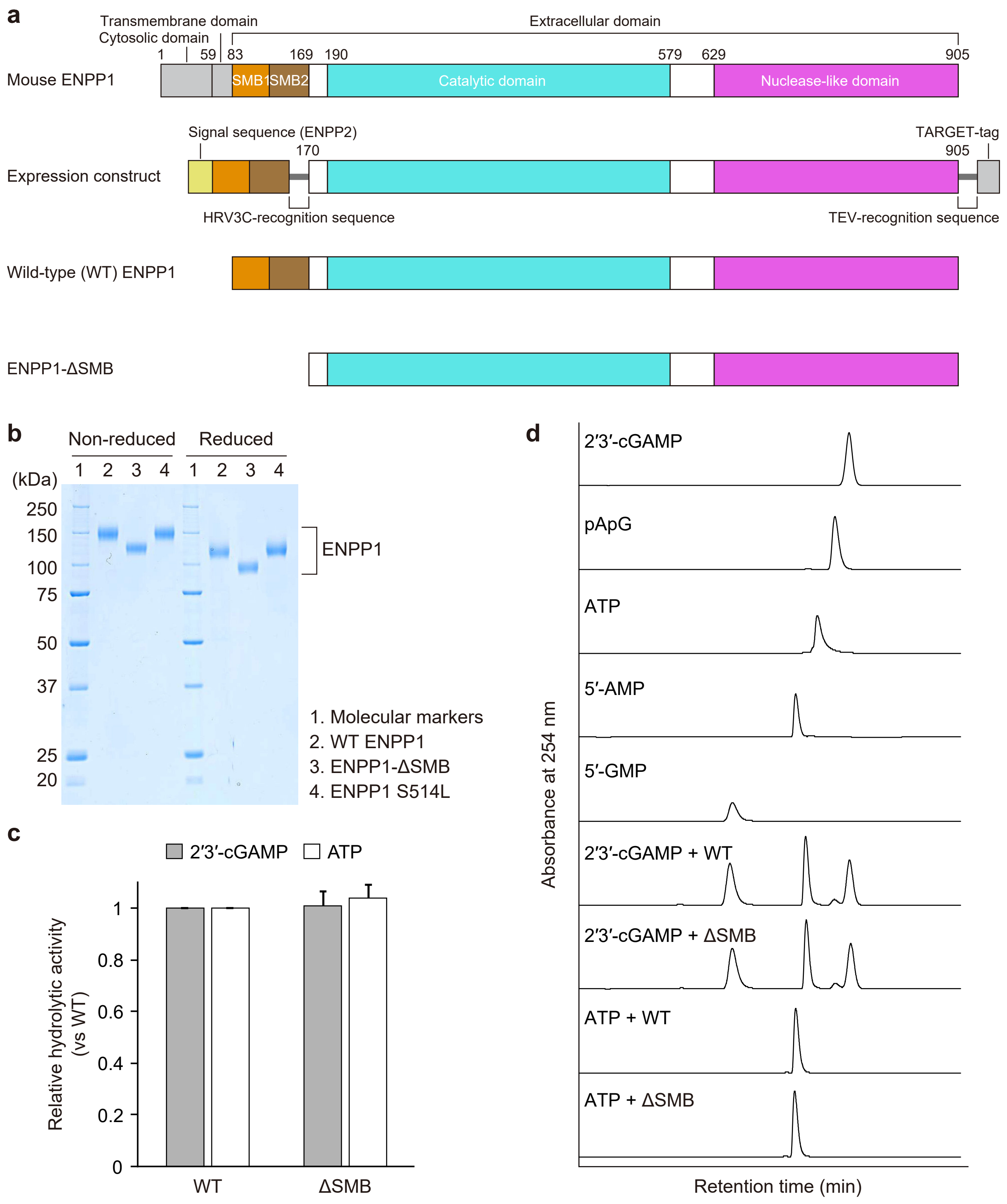

Supplementary Figure 1 | ENPP1 constructs.

a ENPP1 proteins used for the functional and structural analyses. ENPP1 contains the N-terminal cytosolic domain, the transmembrane domain and the extracellular domain. The extracellular domain comprises the two SMB domains (SMB1 and SMB2), the catalytic domain and the nuclease-like domain.

b ENPP1 proteins used for the *in vitro* hydrolysis assay. The purified ENPP1 proteins were analyzed by SDS-PAGE under reduced or non-reduced conditions. The gel was stained with SimplyBlue Safestain.

c Hydrolytic activities of wild-type (WT) ENPP1 and ENPP1-ΔSMB toward 2′3′-cGAMP or ATP. The purified ENPP1 protein (200 nM) was incubated with the nucleotide (500 μM) for 10 min at 37°C, and the mixture was analyzed by reverse-phase HPLC using a C18 column. Data are means ± s.d. (*n* = 3).

d Elution profiles of the standards (2′3′-cGAMP, ATP, pApG, 5′-AMP and 5′-GMP) and the reaction mixtures in the reverse-phase HPLC analyses.

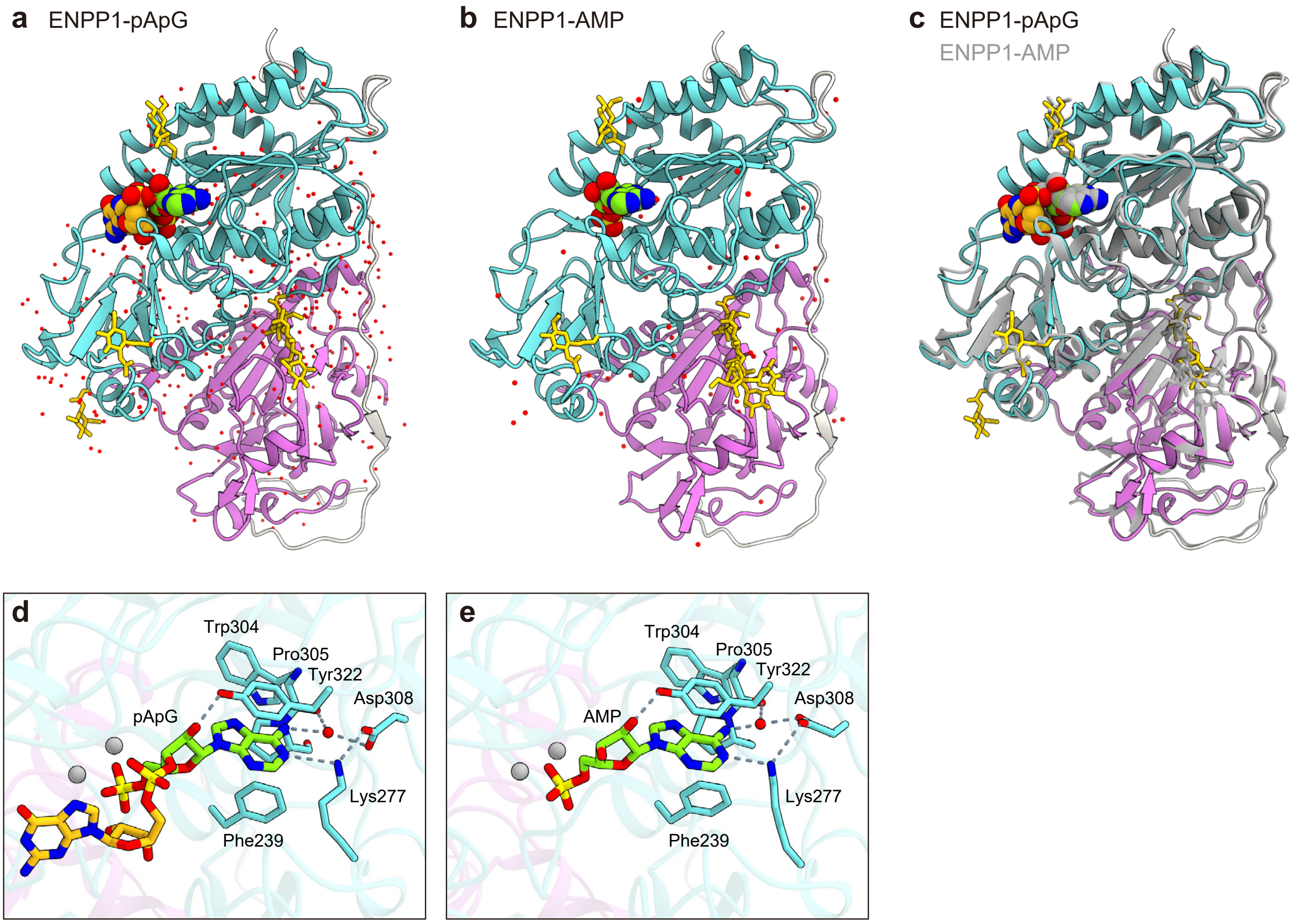

Supplementary Figure 2 | Structural comparison.
a, b Overall structures of ENPP1 in complex with pApG (**a**) and AMP (PDB 4GTW) (**b**). The N-linked sugars and water molecules are shown as yellow sticks and red spheres, respectively.
c Superimposition of ENPP1-pApG and ENPP1-AMP (PDB 4GTW).
d, e Active sites of ENPP1-pApG (**d**) and ENPP1-AMP (PDB 4GTW) (**e**). The zinc ions are shown as gray spheres.

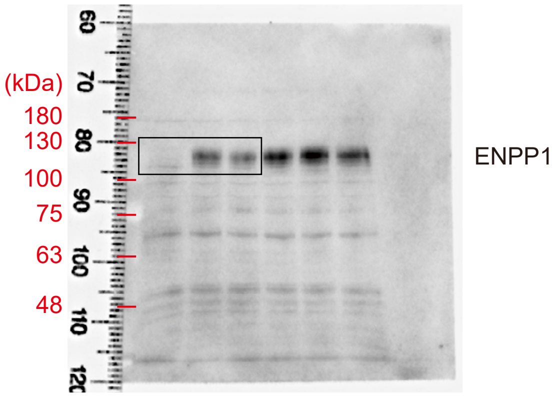

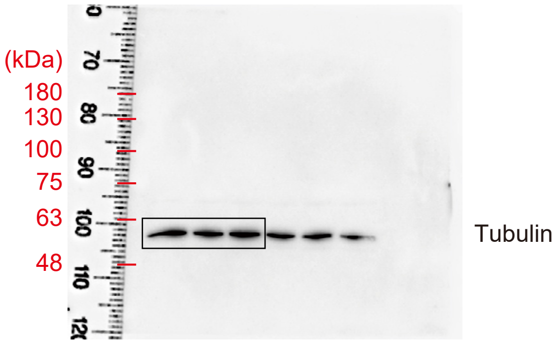

Supplementary Figure 3 | Uncropped images of immunoblots from Figure 3b.

Supplementary Table 1 -Primers used in this study

T238A_F	CTATGTACCCTACCAAGGCCTTTCCCAATCATTAC
T238A_R	GTAATGATTGGGAAAGGCCTTGGTAGGGTACATAG
